# Increase of *c-FOS* promoter transcriptional activity by the dual leucine zipper kinase

**DOI:** 10.1007/s00210-023-02401-z

**Published:** 2023-01-26

**Authors:** Kyra-Alexandra Köster, Jorge Duque Escobar, Anja Fietkau, Regina Toledo, Elke Oetjen

**Affiliations:** 1 Department of Clinical Pharmacology and Toxicology, University Medical Center Hamburg-Eppendorf, Martinistr. 52, 20246 Hamburg, Germany; 2 DZHK Standort Hamburg, Kiel, Lübeck, Germany; 3 Institute of Pharmacy, University of Hamburg, Bundesstr. 45, 20146 Hamburg, Germany

**Keywords:** Dual leucine zipper kinase, c-FOS, Promoter analysis, Genome-edited HIT cell line

## Abstract

The dual leucine zipper kinase (DLK) and the ubiquitously expressed transcription factor c-FOS have important roles in beta-cell proliferation and function. Some studies in neuronal cells suggest that DLK can influence c-FOS expression. Given that c-FOS is mainly regulated at the transcriptional level, the effect of DLK on *c-FOS* promoter activity was investigated in the beta-cell line HIT. The methods used in this study are the following: Luciferase reporter gene assays, immunoblot analysis, CRISPR-Cas9-mediated genome editing, and real-time quantitative PCR. In the beta-cell line HIT, overexpressed DLK increased *c-FOS* promoter activity twofold. Using 5′-,3′-promoter deletions, the promoter regions from − 348 to − 339 base pairs (bp) and from a − 284 to − 53 bp conferred basal activity, whereas the promoter region from − 711 to − 348 bp and from − 53 to + 48 bp mediated DLK responsiveness. Mutation of the cAMP response element within the promoter prevented the stimulatory effect of DLK. Treatment of HIT cells with KCl and the adenylate cyclase activator forskolin increased c-FOS promoter transcriptional activity ninefold. Since the transcriptional activity of those promoter fragments activated by KCl and forskolin was decreased by DLK, DLK might interfere with KCl/forskolin-induced signaling. In a newly generated, genome-edited HIT cell line lacking catalytically active DLK, c-Fos mRNA levels were reduced by 80% compared to the wild-type cell line. DLK increased c-FOS promoter activity but decreased stimulated transcriptional activity, suggesting that DLK fine-tunes c-FOS promoter-dependent gene transcription. Moreover, at least in HIT cells, DLK is required for FOS mRNA expression.

**Supplementary Information:**

The online version contains supplementary material available at 10.1007/s00210-023-02401-z.

## Introduction

The mammalian dual leucine zipper kinase (DLK; mitogen-activated protein 3 kinase 12, MAP3K12) and the closely related leucine zipper kinase (LZK; MAP3K13) share 90% amino acid sequence identity within their enzymatic and their leucine zipper domains. Both are highly conserved orthologues of DLK/Wallenda (*in D. melanogaster*) and DLK-1 (in *C.* *elegans*) hinting at an important role of these kinases (Jin and Zheng 2019). Indeed, mice lacking DLK die perinatally (Hirai et al. 2006), yet, the ablation of DLK in adult mice or of LZK results in no gross phenotypic abnormalities (Pozniak et al. 2013; Chen et al. 2016). Thus, despite their homology, DLK and LZK have both, overlapping and different, functions. Acting as a MAP3K, DLK mainly phosphorylates and activates the MAP2K MKK4 and 7 leading to the phosphorylation of the MAPK c-Jun N-terminal kinase (JNK) (Jin and Zheng 2019). Recruitment to the scaffold protein JNK interacting protein/islet brain 1 (JIP/IB1) keeps monomeric DLK inactive. Upon phosphorylation of JIP/IB1 DLK dissociates from this scaffold protein, homodimerizes via its leucine zipper, and becomes autophosphorylated and enzymatically active (Nihalani et al. 2007). Phosphorylation of DLK itself on Ser-302 (in murine DLK) either through autophosphorylation, by JNK or by protein kinase A (PKA) is required for DLK activity and can activate downstream kinases (Huntwork-Rodriguez et al. 2013; Hao et al. 2016; Börchers et al. 2017). Accordingly, phosphatases interfere with DLK activity as well (Asghari Adib et al. 2018). Preventing the interaction of DLK with the calcium/calmodulin-dependent phosphatase calcineurin enforces DLK-dependent JNK activation (Duque Escobar et al. 2021). Additionally, depending on its subcellular localization, DLK exerts distinct functions, thus contributing to compartmentalized signaling (Holland et al. 2016; Wallbach et al. 2016; Asghari Adib et al. 2018).

DLK is clearly required in neuronal development and postnatal pancreatic beta-cell proliferation (Hirai et al. 2006; Jin and Zheng 2019; Tenenbaum et al. 2020). The ubiquitously expressed transcription factor c-Fos is an important regulator of beta-cell proliferation and function: in the beta-cell lines INS1 and MIN6 c-fos mRNA is increased in a synergistic manner by glucose-induced calcium influx and by GLP-1-dependent increase in intracellular cAMP (Susini et al. 1998; Josefsen et al. 1999). This stimulatory effect is at least in part mediated by the cAMP response element (CRE) within the c-fos promoter (Susini et al. 2000). In addition, palmitate and oleate induced c-fos mRNA in INS1 and MIN6 cells (Roche et al. 1999; Busch et al. 2002). In a rat beta-cell line, the transcription factor NK6 homeobox 1 (NKX6.1) increased c-Fos expression resulting in enhanced Nr4a1, Nr4a3, and VGF expression with beta-cell proliferation and glucose-induced insulin secretion (Ray et al. 2016). Studies in the insulin-secreting beta-cell line HIT showed that DLK activated by diabetic risk factors reduces beta-cell function and mass, thereby contributing to the pathogenesis of diabetes mellitus (Plaumann et al. 2008; Stahnke et al. 2014; Wallbach et al. 2016; Börchers et al. 2017; Duque Escobar et al. 2021). The inhibition of calcium-stimulated CRE/CRE binding protein (CREB)-dependent gene transcription by DLK might contribute to the apoptosis-inducing effect of the kinase (Oetjen et al. 2006; Phu et al. 2011; Wallbach et al. 2016). In embryonal stem cell-derived human neurons, the most important genetic risk factor for AD, the apolipoprotein E4 (ApoE4), after binding to its ApoE receptor activated DLK with subsequent activation of MKK7 and extracellular regulated kinase1/2 (ERK1/2). Activated ERK1/2 in turn phosphorylated the transcription factor c-FOS resulting in enhanced transcriptional activity of the amyloid-beta precursor protein (*App*) promoter and increased amyloid-beta levels (Huang et al. 2017). Furthermore, after induction of stress in cultured embryonic dorsal root ganglion (DRG) by the withdrawal of nerve growth factor (NGF) inhibition of DLK decreased c-fos mRNA (Larhammar et al. 2017); after sciatic nerve transection, a downregulation of Fos in the DRG of conditional DLK-KO mice was observed (Shin et al. 2019). These studies in neuronal cells and tissue demonstrate that DLK influences c-FOS activity and expression. Since c-Fos is mainly regulated at the transcriptional level (Alfonso-Gonzalez and Riesgo-Escovar 2018), in the present study the effect of DLK on the transcriptional activity of the *c-FOS* promoter was investigated in HIT beta-cells.

## Material and methods

### Plasmids

All expression vectors for DLK wild-type (WT) and its diverse mutants (K185A, NLS, NES, V364A) have been described before (Wallbach et al. 2016; Duque Escobar et al. 2021). The luciferase reporter gene − 711 c-fosLuc has been described before (Eckert et al. 1996), and the plasmid − 711 CREmut *c-FOS*Luc was generated by primerless PCR, destroying the CREB binding-site (kind gift of Annette Masuch, Göttingen). The 5′- and 3′-deletions of − 711 c-fosLuc were generated by PCR using the primers listed in Tab. 1 of Supplementary Information (SI). The PCR fragments were cloned into HindIII/XhoI sites of the plasmids pXP2Luc or pT81Luc for the 3′-deletions, respectively. All constructs were verified by sequencing.

### Cell culture and transient transfection

Hamster insulinoma tumor cells (HIT-T15) were cultured in RPMI 1640 medium supplemented with 10% fetal bovine serum, 5% horse serum, 100 units/ml penicillin, and 100 µ/ml streptomycin (Heinrich et al. 2013). Cells were transiently transfected in six-well plates by Metafectene (Biontex, Munich, Germany) according to the manufacturer’s protocol with 1 µg of DNA of the reporter gene. Co-transfections were carried with a constant amount of DNA. To check for transfection efficiency, 0.2 µg DNA/well of an expression vector for a green fluorescent protein under the control of the cytomegalovirus promoter was co-transfected. When indicated, cells were treated with KCl (40 mM) and/or forskolin (10 µM) for 6 h or with GNE-3511 (1 µM) (Cayman Chemical Company, MI, USA) for 8 h, cells were harvested 48 h after transfection. Luciferase activity was measured as described (Heinrich et al. 2013).

### Generation of HIT-K185A cell line

The HIT cell line was genome-edited by the clustered regularly interspaced short palindromic repeats (CRISPR)/Cas9 method using the plasmid-based approach (Ran et al. 2013). Briefly, cells were transiently nucleofected with the plasmid pSpCas9(BB)-2A-GFP (Addgene # 48,138), in which the specific sgRNA was cloned into the Bbs1 site, and the single-stranded homology-directed DNA repair template (HDR template; Eurofins, MWG) (SI, Tab. 1) using the SE Cell Line 4D Nucleofector X Kit L (Lonza Bioscience, Basel, Switzerland) and the Amaxa 4D nucleofector (program DS 150). After nucleofection, cells were treated with SCR-7 (1 µM) (Selleckchem; #S7742; Houston, TX, USA) to increase the efficiency of the homology-directed repair (Chu et al. 2015; Maruyama et al. 2015). After 48 h, GFP-positive cells were sorted by fluorescence-activated cell sorting (BD FACS Aria Illu) directly into 96 well plates and grown in fresh and conditioned HIT cell medium (1:1). After clonal expansion for three to 4 weeks, genomic DNA of the cell clones was isolated and further analyzed by restriction fragment length polymorphism using the newly generated MscI restriction enzyme site and sequencing. Additionally, the fragments containing the ten most likely off-targets for the chosen sgRNA as predicted by the CRISPOR tool (Haeussler et al. 2016) were sequenced. Mutations in these probable off-targets were not detected. This newly generated cell line was named HIT-K185A.

### RNA extraction and qPCR

Total RNA from HIT WT and HIT-K185A cells was isolated by RNAzol RT (Sigma-Aldrich, Steinhein, Germany) followed by a cleaning step (Monarch RNA clean-up kit (New England Biolabs, Beverly, MA, USA) according to the manufacturer’s protocol. RNA concentration was determined by NanoDrop™ 2000c (Thermo Scientific, MA, USA) and was reverse transcribed using the High Capacity cDNA reverse transcription kit (Applied Biosystems, Thermo Fischer, Vilnius, Lithuania). *DLK* and *Fos* mRNA analysis was performed using a gene expression master mix and the following gene expression assays (Applied Biosystems, MA, USA): *Fos* (Mm00487425_m1), *Actb* (Mm02619580_g1), and *Hprt* (Mm03024075_m1). The similarity between mouse and hamster *Fos* is 91.68%. For quantification, QuantStudio 7 Flex (Applied Biosystems, MA, USA) was used. Expression of *Dlk* and *Fos* mRNA was normalized to the geometrical mean of murine *Actb* and *Hprt* using the formula 2^−ΔΔCT^.

### Immunoblot

HIT WT cells and HIT-K185A cells were harvested in lysis buffer (Oetjen et al. 2007), and equal amounts of protein were subjected to SDS-PAGE and immunoblot analysis using antibodies against DLK (1:3000) (Oetjen et al. 2006) or (1:3000; GTX124127) (GeneTex, CA, USA), GAPDH (1:60,000; #sc-32233; 6C5) (Santa Cruz, Heidelberg, Germany) and α-Tubulin (1:1000; #2125) (Cell Signaling Technology, Beverly, MA, USA) were performed. The immunoreactive bands were visualized using ECL or ECLmax (for DLK detection in HIT-K185A cells) (BioRad Laboratories, München, Germany) and a chemiluminescence imaging system (ChemiDoc™ Touch Imaging System, BioRad Laboratories, München, Germany). Densitometric evaluation was performed using ImageLab 6.0 analysis software (BioRad Laboratories, München, Germany). To determine DLK half-time in the HIT WT and HIT-K185A cell line, cells were treated with cycloheximide (5 µg/ml, dissolved in DMSO) (Sigma-Aldrich, Steinheim, Germany) at the indicated time points before harvest. When indicated, cells were transiently transfected with expression vectors for DLK wild-type or its mutants.

### Statistics

Data are expressed as mean ± standard error of the mean (SEM). Statistical analysis using Prism 8.0 (Graphpad Software Inc., CA, USA) was as stated in the legends.

## Results

### Effect of DLK on the transcriptional activity of the c-*FOS* promoter

**Fig. 1:**
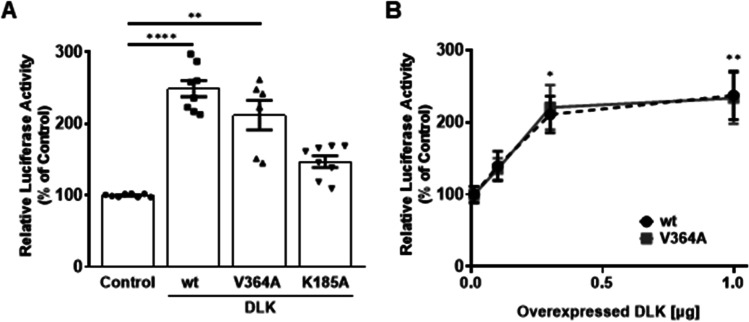
Effect of DLK on the transcriptional activity of the *c-FOS* promoter. **A** The luciferase reporter gene under control of the *c-FOS* promoter from − 711 to + 48 bp was transiently cotransfected with expression vectors for DLK wt, DLK V364A, and DLK K185A into HIT cells. Cells were harvested 48 h after transfection and luciferase activity was determined. Values present the ratio of luciferase and GFP activity relative to the activity of the *c-FOS* promoter in the absence of overexpressed DLK or its mutants set as 100%. Values are means ± SEM of four different experiments, each done in duplicate. **p ≤ 0.01; ****p ≤ 0.0001 vs. control, Kruskal–Wallis followed by Dunn’s multiple comparisons. **B** DLK wt and DLK V364 increase *c-FOS* promoter activity to similar extents. The luciferase reporter gene under control of the *c-FOS* promoter was transiently cotransfected with increasing amounts of the expressions vector for DLK wt (black dots) and DLK V364A (grey squares). Values present the ratio of luciferase and GFP activity relative to the activity of the *c-FOS* promoter in the absence of overexpressed DLK wt or DLK V364A set as 100%. Values are means ± SEM of four different experiments, each done in duplicate. *p ≤ 0.05; **p ≤ 0.01 vs control, Kruskal–Wallis followed by Dunn’s multiple comparisons

To investigate the effect of DLK on the transcriptional activity of the human *c-FOS* promoter, a luciferase reporter gene under the control of the human *c-FOS* promoter (from –711 to + 48 bp) was transiently co-transfected into HIT cells with expression vectors for DLK wildtype (WT), the calcineurin binding-deficient mutant (V364A) and the ATP binding-deficient mutant (K185A). Both, DLK WT and DLK V364A enhanced *c-FOS* promoter transcriptional activity approximately twofold while DLK K185A had no effect (Fig. 1A). Taken into account that DLK V364A phosphorylated JNK to a higher degree than DLK WT, it was tested whether DLK V364A was more active than DLK WT to increase transcriptional activity (Duque Escobar et al. 2021). Increasing amounts of expression vectors for DLK WT and the V364A were transiently co-transfected with the *c-FOS* promoter-luciferase gene. Similar amounts of the expression vectors for DLK WT and DLK V364A resulted in a similar increase of *c-FOS* promoter-dependent gene transcription (Fig. 1B). Considering that the autophosphorylation of DLK WT and DLK V364A was alike (Duque Escobar et al. 2021), these data suggest, that DLK-caused activation of the *c-FOS* promoter was independent of the DLK downstream kinase JNK.

**Fig. 2:**
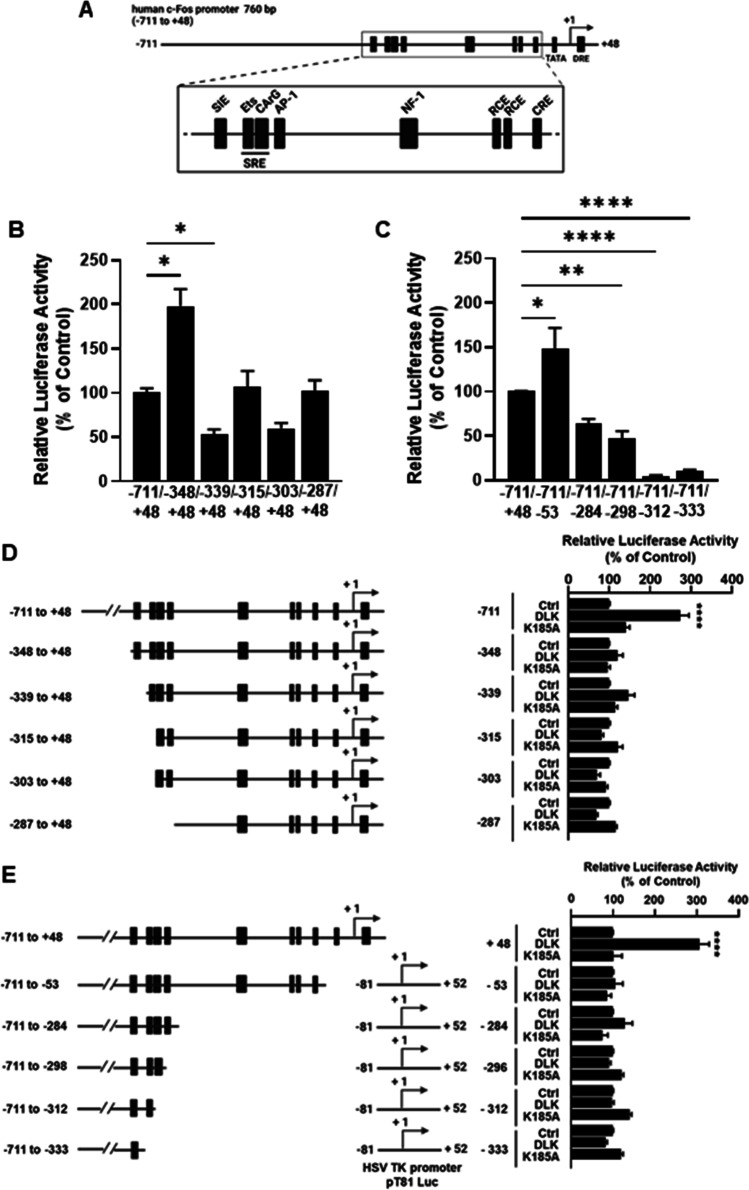
Effect of DLK on 5′- and 3′-deletions of the *c-FOS* promoter. **A** Scheme of the c-FOS promoter. The proximal promoter region is enlarged to better depict the diverse binding-sites for the transcription factors. **B** Transcriptional activities of the 5′-deleted promoter fragments. Values represent the ratio of luciferase and GFP activity relative to the activity of the *c-FOS* promoter from − 711 to + 48 bp set as 100%. Values are means ± SEM of three to four different experiments, each done in duplicate. *p ≤ 0.0001, Kruskal–Wallis test and two-stage linear step-up procedure of Benjamini, Krieger, and Yekutieli. **C** Transcriptional activities of the 3′-deleted promoter fragments. Values represent the ratio of luciferase and GFP activity relative to the activity of the *c-FOS* promoter from − 711 to + 48 bp set as 100%. Values are means ± SEM of five different experiments, each done in duplicate. *p ≤ 0.01 **p ≤ 0.005 ****p ≤ 0.0001, one-way ANOVA and Dunnett’s multiple comparisons test. **D** Effect of DLK on 5′-deletions of the *c-FOS* promoter. Left panel, scheme of the 5′-deletions. Right panel, transcriptional activities of the 5′-deleted promoter fragments with overexpressed DLK wt or DLK K185A mutant. Values represent the ratio of luciferase and GFP activity relative to the activity of the respective 5′-deleted *c-FOS* promoter fragment in the absence of overexpressed DLK set as 100%. Values are means ± SEM of three to four different experiments, each done in duplicate. ****p ≤ 0.0001, Kruskal–Wallis and two-stage linear step-up procedure of Benjamini, Krieger, and Yekuteli. **E** Effect of DLK on 3′-deletions of the *c-FOS* promoter. Left panel, scheme of the 3′-deletions. Right panel, transcriptional activities of the 3′-deleted promoter fragments with overexpressed DLK wt or DLK K185A mutant. Values represent the ratio of luciferase and GFP activity relative to the activity of the respective 3′- deleted *c-FOS* promoter fragment in the absence of overexpressed DLK set as 100%. Values are means ± SEM of five different experiments, each done in duplicate. *p* ≤ 0.0001, One-way ANOVA followed by Tukey’s multiple comparisons

To identify a DLK-responsive element within the *c-FOS* promoter the effect of the kinase on the transcriptional activity of 5′- and 3′-deleted promoter fragments was studied. The 5′-deletion of 363 bp upstream of − 348 bp increased the transcriptional activity of the *c-FOS* promoter app. twofold (2.0 ± 0.08; *n* = 4), indicating that this promoter region represses *c-FOS* dependent gene transcription. Further 5′-deletion of the binding-site for the signal transducer and activator of transcription (STAT) transcription factors severely decreased *c-FOS* promoter activity and was not re-established by the deletion of additional bp (Fig. 2B). For the 3′-deletions of the *c-FOS* promoter, the proximal minimal promoter fragment of the *c-FOS* gene was substituted by the minimal promoter of the herpes simplex thymidine kinase. Deletion of the base pairs from − 52 to + 48 containing the binding-site for the transcription factor downstream regulatory element antagonist modulator (DREAM) increased the basal promoter activity. Further 3′-deletions up to − 298 bp reduced the transcriptional activity and deletions beyond − 312 bp decreased the transcriptional activity by 95% (Fig. 2C). These data show that the 5′-deletion up to − 339 bp and 3′-deletion up to − 284 bp decreased the transcriptional activity of the *c-FOS* promoter, suggesting that multiple transcription binding sites contribute to *c-FOS* promoter activity. Overexpression of DLK increased *c-FOS* promoter activity approximately 2.7-fold. This stimulatory effect of DLK was lost in all 5′-deleted promoter fragments (Fig. 2D). In addition, the stimulatory effect of DLK on *c-FOS* promoter activity was lost when the proximal promoter fragment from − 52 to + 48 bp was deleted (Fig. 2E). Thus, the poorly characterized region ranging from − 711 to − 348 bp and the proximal promoter containing the downstream regulatory element (DRE) bound by DREAM cooperatively confer the stimulatory effect of DLK to the *c-FOS* promoter.

### Effect of DLK on stimulated transcriptional activity of the c-FOS promoter

**Fig. 3:**
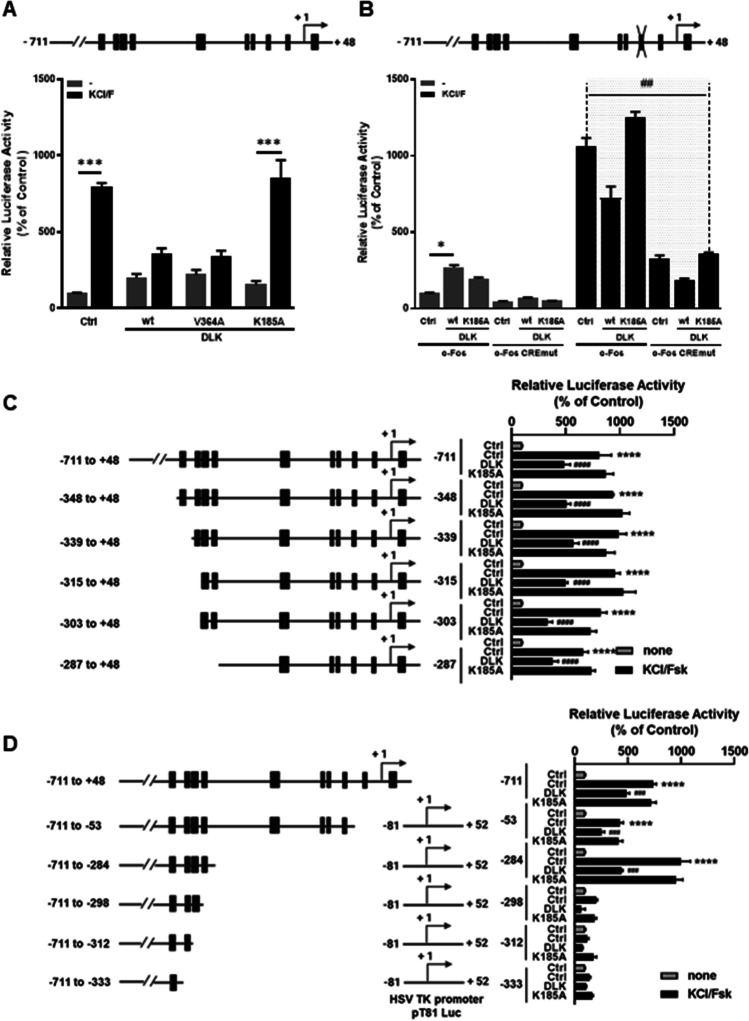
Effect of DLK on stimulated transcriptional activity of the *c-FOS* promoter. **A** The luciferase reporter gene under control of the *c-FOS* promoter was transiently co-transfected with expression vectors for DLK and its mutants. Cells were treated 6 h prior to harvest with KCl (40 mM) and forskolin (10 µM) as indicated. Values represent the ratio of luciferase and GFP activity relative to the activity of the *c-FOS* promoter from − 711 to + 48 bp without treatment set as 100%. Values are means ± SEM of three different experiments, each done in duplicate. ***p ≤ 0.001. Two-way ANOVA followed by Sidak’s multiple comparison test. **B** The luciferase reporter genes under control of the *c-FOS* promoter or the CREmut *c-FOS* promoter, respectively, were transiently co-transfected with DLK wt or the K185A mutant and treated 6 h prior to harvest with KCl and forskolin. Values represent the ratio of luciferase and GFP activity relative to the activity of the *c-FOS* promoter from − 711 to + 48 bp without treatment set as 100%. Values are means ± SEM of three different experiments, each done in duplicate. The grey shaded area denotes a difference between the transcriptional activities of the c-*FOS* promoter and the CREmut c-*FOS* promoter in the respective treatment group. *p ≤ 0.05, ^##^*p* ≤ 0.01. Two-way ANOVA followed by Tukey’s multiple comparisons. **C** Effect of DLK on stimulated transcriptional activity of 5′-deletions of the *c-FOS* promoter. The diverse luciferase reporter genes were transiently co-transfected with DLK wt or DLK K185A and treated 6 h prior to harvest with KCl and forskolin. Values represent the ratio of luciferase and GFP activity relative to the activity of the 5′-deleted *c-FOS* promoter fragments in the absence of treatment set as 100%. Values are means ± SEM of three different experiments, each done in duplicate. ****p ≤ 0.0001 respective control (Ctrl, grey column) vs. stimulated control (Ctrl, black column), ^####^*p* ≤ 0.0001 respective stimulated control (Ctrl, black column) vs. stimulated activity in the presence of DLK (DLK), One-way ANOVA followed by Tukey’s multiple comparisons. **D** Effect of DLK on stimulated transcriptional activity of 3′-deletions of the *c-FOS* promoter. The diverse luciferase reporter genes were transiently co-transfected with DLK wt or DLK K185A and treated 6 h prior to harvest with KCl and forskolin. Values represent the ratio of luciferase and GFP activity relative to the activity of the 3′-deleted *c-FOS* promoter fragments in the absence of treatment set as 100%. Values are means ± SEM of four different experiments, each done in duplicate. ****p ≤ 0.0001 respective control (Ctrl, grey column) vs. stimulated control (Ctrl, black column), ^###^*p* ≤ 0.001 respective stimulated control (Ctrl, black column) vs. stimulated activity in the presence of DLK (DLK), one-way ANOVA followed by Tukey’s multiple comparisons

The transcriptional activity and expression of *c-FOS* are regulated by many diverse stimuli like stress signals, membrane depolarization, and various growth factors increasing the levels of second messengers like cyclic AMP (cAMP) and calcium among others (Kovács 1998; Qiu and Ghosh 2008; Langfermann et al. 2019). To investigate whether DLK interferes with stimulated *c-FOS*-dependent transcription, HIT cells were treated with KCl to induce membrane depolarization with calcium influx and with the adenylate cyclase activator forskolin increasing intracellular cAMP and indirectly calcium. The combined stimulus enhanced *c-FOS-*dependent transcriptional activity 7.9 ± 0.1-fold (*n* = 6) (Fig. 3A). In the presence of DLK WT or DLK V364A, stimulated *c-FOS* transcriptional activity was severely diminished, whereas the kinase-dead DLK mutant had no effect on *c-FOS*-dependent gene transcription (Fig. 3A). The transcription factor cAMP response element binding protein (CREB) bound to the cAMP response element (CRE) is well known to confer membrane depolarization- and forskolin-induced transcriptional activity. In addition, DLK regulates CRE/CREB-dependent transcription (Oetjen et al. 2006; Phu et al. 2011; Wallbach et al. 2016; Duque Escobar et al. 2021). To study whether the CRE mediates DLK or KCl/forskolin-induced transcriptional activity to *c-FOS*, the CRE within the promoter was mutated to prohibit CREB binding. Mutation of the CRE did not interfere with the basal transcriptional activity of the *c-FOS* promoter and somewhat decreased its KCl/forskolin-stimulated activity (Fig. 3B), suggesting that in addition to the CRE other DNA binding-sites mediate KCl/forskolin-induced signaling to the *c-FOS* promoter. Yet, mutation of the CRE within the *c-FOS* promoter prevented the stimulatory effect of DLK, but DLK still decreased KCl/forskolin-induced *c-FOS* transcriptional activity (Fig. 3B). The transcriptional activity of all 5′-deleted promoter fragments was induced by KCl/forskolin treatment and DLK inhibited these enhanced transcriptional activities; the transcriptional activities of those 3′-deleted promoter fragments enhanced by KCl/forskolin was decreased by DLK (Fig. 3C and D). These data suggest that DLK exerts distinct functions on *c-FOS* promoter transcriptional activity: the kinase increases *c-FOS* promoter-dependent gene transcription, whereby the CRE within the promoter and the promoter fragments from − 53 to + 48 bp, and from − 711 to − 348 bp contribute to the DLK response. Furthermore, as long as KCl/forskolin enhance *c-FOS* promoter-dependent transcriptional activity, this activity is decreased by DLK, suggesting that DLK interferes with KCl/forskolin signal transduction.

### Effect of DLK on c-*Fos* mRNA levels

**Fig. 4:**
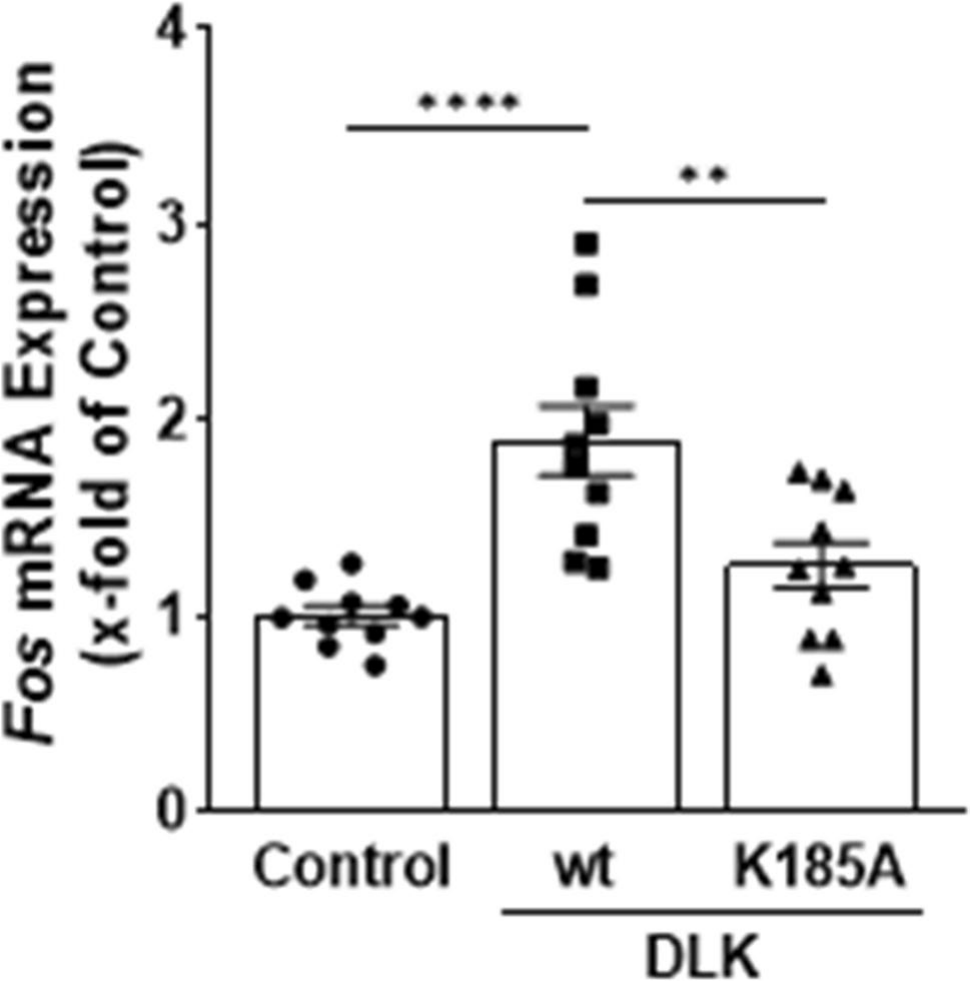
Effect of DLK on *c-fos* mRNA level. HIT cells were transiently transfected with DLK WT or DLK K185A as indicated. Cells were harvested 48 h after transfection and mRNA was isolated. The amount of *c-fos* mRNA level was determined by qPCR normalizing the CT-values of *Fos* to the geometrical mean of *Actb*/*Hprt*. Data are presented as fold change compared to cells transfected with Bluescript (empty vector, control), set as 1. Values are means ± SEM of ten different experiments. One-way ANOVA followed by Tukey’s multiple comparison test. **p < 0.01, ****p < 0.001

**Fig. 5:**
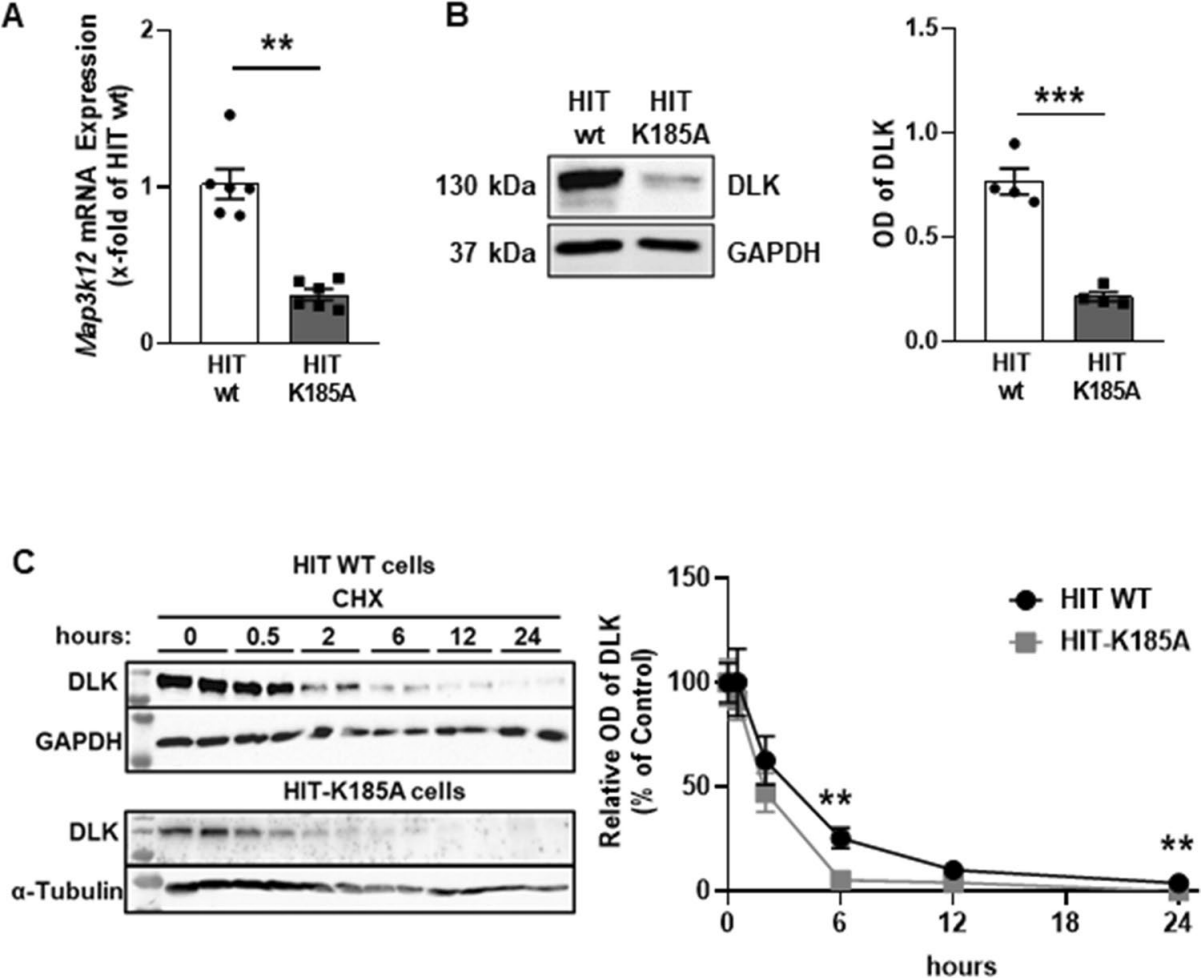
Brief characterization of the new genome-edited HIT cell line HIT-K185A. **A** Comparison of DLK mRNA level in wild-type (WT) and genome-edited HIT-K185A cells. Cells from the different cell lines were harvested and *dlk* mRNA levels were determined by qPCR. Values present relative *DLK* mRNA levels set as 1 in the WT HIT cells and are means of six different experiments. **p ≤ 0.01; unpaired *t*-test. **B** Reduced DLK protein in HIT-K185A cells. Left panel, typical immunoblot; right panel, quantitative evaluation. HIT WT and HIT-K185A cells were harvested, an immunoblot using an antibody against DLK was performed and the optical density of the respective bands was determined. Per lane, 35 µg of protein were loaded. Values present relative DLK protein levels set as 1 in HIT WT cells and are means of four experiments. ***p ≤ 0.001, unpaired *t*-test. **C** DLK K185A is less stable than DLK wild-type. Left panel, typical immunobots, Right panel, quantitative evaluation. Cells from the HIT WT and the HIT-K185A line were treated with cycloheximide (5 µg/ml per well) at the indicated time points before harvest and subjected to immunoblot analysis and the optical density of the respective bands was evaluated. For the immunoblot, 50 µg of protein per lane were loaded. Left panel, typical blot; right panel, quantitative evaluation. Values present the relative DLK protein levels in the absence of cycloheximide set as 100% in each cell line and are means of three experiments, each done in duplicate. **p ≤ 0.01, Mann–Whitney test

**Fig. 6:**
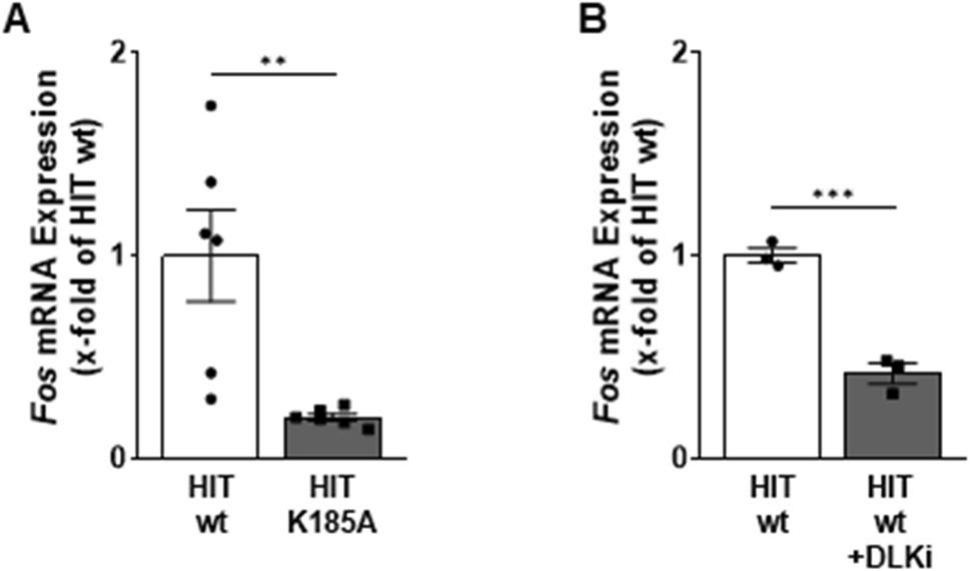
Effect of reduced DLK activity on *c-fos* mRNA level. **A** Decrease of *c-fos* mRNA level in genome-edited HIT cells lacking active DLK. After harvest, mRNA was extracted and the level of *c-fos* mRNA was determined by qPCR normalizing the CT-values of *Fos* to the geometrical mean of *Actb*/*Hprt*. Values are relative to the mRNA level in HIT WT cells, set as 1. Values are means ± SEM of six different experiments. **p < 0.01, Mann–Whitney test. **B** HIT WT cells were treated with GNE-3511 (1 µM) for 8 h. After harvest, mRNA was extracted and the level of *c-fos* mRNA was determined by qPCR normalizing the CT-values of *Fos* to the geometrical mean of *Actb*/*Hprt*. Values are relative to the mRNA level in the untreated control, set as 1. Values are means ± SEM of three different experiments, unpaired *t*-test; ***p < 0.001

To investigate whether DLK increases *c-Fos* mRNA level, HIT cells were transiently transfected with the expression vectors for DLK WT and K185A. DLK WT but not K185A slightly increased *c-Fos* mRNA level (Fig. 4). Since the transfection efficiency in HIT cells is low, these data might underestimate the effect of DLK on *c-Fos* mRNA level. Therefore, a new HIT cell line was generated, in which the lysine within the ATP-binding pocket of DLK was mutated to alanine by CRISPR/Cas9-mediated genome editing. Initial characterization of this newly generated cell line HIT-K185A revealed a reduction of *Dlk* mRNA levels (Fig. 5A). In addition, the protein content of the mutated DLK was severely decreased (Fig. 5B). Prevention of protein neosynthesis by cycloheximide revealed a faster degradation of DLK in the HIT-K185A cell line than in HIT WT cells with DLK half-times of 2.0 and 2.5 h, respectively (Fig. 5C). These findings are consistent with the notion that non-phosphorylated DLK has reduced protein stability (Huntwork-Rodriguez et al. 2013). In this new cell line lacking enzymatically active DLK, the *c-Fos* mRNA level was decreased by 80%, suggesting that DLK is required for *c-Fos* expression (Fig. 6A). In line, treatment of HIT WT cells for 8 h with the DLK inhibitor GNE-3511 (Patel et al. 2015; Le Pichon et al. 2017) decreased *c-Fos* mRNA level by 60% (Fig. 6B), whereas 1-h and 12-h treatment had no effect on *c-Fos* mRNA levels (not shown). Thus, DLK enhances c-FOS activity through phosphorylation of the protein (Huang et al. 2017) and through *c-FOS* gene transcription (this study).

## Discussion

The transcription factor c-FOS is part of the activator protein (AP)-1 formed by heterodimers of members of the FOS and the JUN families. Both groups of proteins are basic region leucine zipper (bZip) proteins, heterodimerizing via their leucine zippers and binding to DNA-sites with the consensus motif 5′-TGA G/C TCA-3′ via their basic region (Alfonso-Gonzalez and Riesgo-Escovar 2018; Bejjani et al. 2019). AP-1 transcription factors are ubiquitously expressed and have been implicated in such opposing functions like cell death and cell proliferation and thus in various diseases among them several inflammatory diseases, cancer, fibrosis, and rejection of transplanted organs (Shaulian and Karin 2002; Durchdewald et al. 2009; Bejjani et al. 2019). Furthermore, through acting on distal enhancers, FOS might be involved in changing the chromatin structure thereby controlling activity-dependent gene programs (Malik et al. 2014; Bejjani et al. 2019).

Phosphorylation on serine/threonine and tyrosine residues regulates c-FOS activity (Huang et al. 2017; Alfonso-Gonzalez and Riesgo-Escovar 2018), but many stimuli like exposure to UV light, the addition of serum, peroxide, mechanical stretching, high glucose, incretins, and calcium influx among others increase the expression of *c-fos* and c-FOS within minutes and very few hours, respectively, making *c-fos* the typical “immediate early gene” (Susini et al. 1998; Josefsen et al. 1999; Roche et al. 1999; Kovács 2008; Alfonso-Gonzalez and Riesgo-Escovar 2018). Considering the short half-time of *c-fos* and c-FOS (approximately 30 min and 2 h, respectively) (Kovács 2008), c-FOS activity is mainly regulated at the transcriptional level. In turn, c-FOS itself regulates the transcriptional activity of many genes, thus contributing to the adaptation of cells to external stimuli (Shaulian and Karin 2002; Durchdewald et al. 2009; Alfonso-Gonzalez and Riesgo-Escovar 2018; Bejjani et al. 2019). For instance, in a rat beta cell line, the transcription factor NKX6.1 increases *Nr4a1*,* Nr4a3*, and *VGF* expression in a *c-fos*-dependent way. Hence, signals increasing the immediate early gene *c-fos* expression can change the expression of several additional genes promoting a long-term adaptation to these signals. Using overexpression of DLK and its enzymatically dead mutant, DLK inhibition by GNE-3511, and a newly generated genome-edited HIT cell line, expressing the kinase-dead DLK mutant, the present study shows that DLK enhanced *c-FOS* promoter activity and is required for *c-Fos* mRNA expression. DLK itself becomes activated by stress and injury signals, ApoE signaling, tumor necrosis factor α, hydrogen peroxide, and inhibitors of calcineurin (Börchers et al. 2017; Huang et al. 2017; Jin and Zheng 2019; Duque Escobar et al. 2021) suggesting that at least some effects elicited by DLK activation are mediated by upregulation of the immediate early gene *c-Fos*.

DLK has dual effects on *c-FOS* promoter activity: the kinase inhibited KCl/forskolin-induced transcription and increased basal promoter activity. These findings indicate that DLK interferes with KCl and/or forskolin-provoked signaling pathways. In HIT cells, KCl treatment resulting in membrane depolarization increases the intracellular calcium concentration. Forskolin might by increasing cAMP levels, activation of PKA, and phosphorylation of the voltage-dependent L-type calcium channel enhance the calcium concentration as well. Calcium then activates calcium-dependent enzymes among them calcineurin. DLK interacts with calcineurin via the phosphatase’s substrate recognition site thereby preventing the access of other calcineurin substrates to the phosphatase (Duque Escobar et al. 2021). Hence, through inhibition of calcineurin activity, DLK might decrease KCl/forskolin-induced *c-FOS* promoter activity. In contrast to its inhibitory action on KCl/forskolin-induced c-FOS promoter activity, DLK increased the basal transcriptional activity of this promoter. Using 5′- and 3′-deletions, different DLK-responsive promoter regions, namely the poorly characterized 5′- promoter region from − 711 to − 348 bp and the 3′-promoter region from − 53 to + 48 bp were identified. Moreover, mutation of the CRE within the promoter abolished the stimulatory effect of DLK. Thus, in contrast to the human insulin gene promoter, the DLK responsiveness of the c-FOS promoter is not conferred by a single transcription factor but by a combination of different transcription factors binding to distinct promoter elements (Stahnke et al. 2014) (this study). This is in line with the finding that multiple elements determine the basal transcriptional activity of the *c-FOS* promoter (Lucibello et al. 1991; Runkel et al. 1991) (this study). Since mutation of the CRE within the promoter abolished the stimulatory effect of DLK, CREB, or other CRE binding proteins might play a crucial role in coordinating the assembly of transcription factors and co-activators to mediate DLK-induced transcriptional activity of the *c-FOS* promoter. Of note, the transcription factor Sp1 through recruitment of the transcription activator BRG1 with the retinoblastoma protein (Rb) and the calcium responsive transactivator CREST regulates calcium-dependent *c-fos* promoter activity (Qiu and Ghosh 2008); MAPK signaling enhanced the recruitment of the transcription factor NF1 to the *c-fos* promoter through phosphorylation of the transcription factor Elk-1 and subsequent interaction with the transcription co-activator p300 (O'Donnell et al. 2008). Thus, *c-FOS* promoter activity is regulated by diverse interactions of transactivator proteins with transcription factors.

DLK exerts many and in part controversial effects concerning neuronal development, neurodegeneration after insults, axon regeneration, neonatal beta-cell proliferation, and beta-cell apoptosis after exposure to diabetic risk factors (Tedeschi and Bradke 2013; Oetjen and Lemcke 2016; Börchers et al. 2017; Jin and Zheng 2019; Tenenbaum et al. 2020; Duque Escobar et al. 2021). Considering the involvement of FOS in many functions and its regulation mainly at the transcriptional level, it is tempting to speculate that at least some of these controversial effects might be explained by the dual effects of DLK on *c-FOS* promoter activity: activation of basal activity and inhibition of calcium- and cAMP-induced activity. DLK thereby contributes to the fine-tuning of c-FOS activation.

## Supplementary Information

Below is the link to the electronic supplementary material.

Supplementary file1 (DOCX 13 KB)

## Data Availability

The datasets generated during and/or analyzed during the current study are available from the corresponding author on reasonable request.

## References

[CR1] Alfonso-Gonzalez C, Riesgo-Escovar JR (2018). Fos metamorphoses: lessons from mutants in model organisms. Mech Dev.

[CR2] AsghariAdib E, Smithson LJ, Collins CA (2018). An axonal stress response pathway: degenerative and regenerative signaling by DLK. Curr Opin Neurobiol.

[CR3] Bejjani F, Evanno E, Zibara K, Piechaczyk M, Jariel-Encontre I (2019). The AP-1 transcriptional complex: Local switch or remote command?. Biochimica et Biophysica Acta (BBA) Rev Cancer.

[CR4] Börchers S, Babaei R, Klimpel C, Duque Escobar J, Schröder S, Blume R, Malik MNH, Oetjen E (2017). TNFα-induced DLK activation contributes to apoptosis in the beta-cell line HIT. Naunyn-Schmiedeberg’s Arch Pharmacol.

[CR5] Busch AK, Cordery D, Denyer GS, Biden TJ (2002). Expression profiling of palmitate- and oleate-regulated genes provides novel insights into the effects of chronic lipid exposure on pancreatic β-cell function. Diabetes.

[CR6] Chen M, Geoffroy CG, Wong HN, Tress O, Nguyen MT, Holzman LB, Jin Y, Zheng B (2016). Leucine Zipper-bearing Kinase promotes axon growth in mammalian central nervous system neurons. Sci Rep.

[CR7] Chu VT, Weber T, Wefers B, Wurst W, Sander S, Rajewsky K, Kühn R (2015). Increasing the efficiency of homology-directed repair for CRISPR-Cas9-induced precise gene editing in mammalian cells. Nat Biotechnol.

[CR8] Duque Escobar J, Kutschenko A, Schröder S, Blume R, Köster K-A, Painer C, Lemcke T, Maison W, Oetjen E (2021). Regulation of dual leucine zipper kinase activity through its interaction with calcineurin. Cell Signal.

[CR9] Durchdewald M, Angel P, Hess J (2009). The transcription factor Fos: a janus-type regulator in health and disease. Histol Histopathol.

[CR10] Eckert B, Schwaninger M, Knepel W (1996). Calcium-mobilizing insulin secretagogues stimulate transcription that is directed by the cyclic adenosine 3',5'-monophosphate/calcium response element in a pancreatic islet beta-cell line. Endocrinology.

[CR11] Haeussler M, Schönig K, Eckert H, Eschstruth A, Mianné J, Renaud J-B, Schneider-Maunoury S, Shkumatava A, Teboul L, Kent J, Joly J-S, Concordet J-P (2016). Evaluation of off-target and on-target scoring algorithms and integration into the guide RNA selection tool CRISPOR. Genome Biol.

[CR12] Hao Y, Frey E, Yoon C, Wong H, Nestorovski D, Holzman LB, Giger RJ, DiAntonio A, Collins C (2016) An evolutionarily conserved mechanism for cAMP elicited axonal regeneration involves direct activation of the dual leucine zipper kinase DLK. Elife Elife 5:e14048. 10.7554/eLife.14048 10.7554/eLife.14048 PMC4896747 27268300

[CR13] Heinrich A, der Heyde ASV, Böer U, Phu DT, Tzvetkov M, Oetjen E (2013). Lithium enhances CRTC oligomer formation and the interaction between the CREB coactivators CRTC and CBP — implications for CREB-dependent gene transcription. Cell Signal.

[CR14] Hirai S-i, Feng Cui D, Miyata T, Ogawa M, Kiyonari H, Suda Y, Aizawa S, Banba Y, Ohno S (2006). The c-Jun N-terminal kinase activator dual leucine zipper kinase regulates axon growth and neuronal migration in the developing cerebral cortex. J Neurosci.

[CR15] Holland SM, Collura KM, Ketschek A, Noma K, Ferguson TA, Jin Y, Gallo G, Thomas GM (2016). Palmitoylation controls DLK localization, interactions and activity to ensure effective axonal injury signaling. Proc Natl Acad Sci U S A.

[CR16] Huang Y-WA, Zhou B, Wernig M, Südhof TC (2017). ApoE2, ApoE3, and ApoE4 Differentially Stimulate APP Transcription and Aβ Secretion. Cell.

[CR17] Huntwork-Rodriguez S, Wang B, Watkins T, Ghosh AS, Pozniak CD, Bustos D, Newton K, Kirkpatrick DS, Lewcock JW (2013). JNK-mediated phosphorylation of DLK suppresses its ubiquitination to promote neuronal apoptosis. J Cell Biol.

[CR18] Jin Y, Zheng B (2019). Multitasking: dual leucine zipper–bearing kinases in neuronal development and stress management. Annu Rev Cell Dev Biol.

[CR19] Josefsen K, Sørensen LR, Buschard K, Birkenbach M (1999). Glucose induces early growth response gene (Egr-1) expression in pancreatic beta cells. Diabetologia.

[CR20] Kovács KJ (1998). Invited review c-Fos as a transcription factor: a stressful (re)view from a functional map. Neurochem Int.

[CR21] Kovács KJ (2008). Measurement of immediate-early gene activation- c-fos and beyond. J Neuroendocrinol.

[CR22] Langfermann DS, Schmidt T, Rössler OG, Thiel G (2019). Calcineurin controls gene transcription following stimulation of a Gαq-coupled designer receptor. Exp Cell Res.

[CR23] Larhammar M, Huntwork-Rodriguez S, Jiang Z, Solanoy H, Sengupta Ghosh A, Wang B, Kaminker JS, Huang K, Eastham-Anderson J, Siu M, Modrusan Z, Farley MM, Tessier-Lavigne M, Lewcock JW, Watkins TA (2017). Dual leucine zipper kinase-dependent PERK activation contributes to neuronal degeneration following insult. eLife.

[CR24] Le Pichon CE, Meilandt WJ, Dominguez S, Solanoy H, Lin H, Ngu H, Gogineni A, Sengupta Ghosh A, Jiang Z, Lee S-H, Maloney J, Gandham VD, Pozniak CD, Wang B, Lee S, Siu M, Patel S, Modrusan Z, Liu X, Rudhard Y, Baca M, Gustafson A, Kaminker J, Carano RAD, Huang EJ, Foreman O, Weimer R, Scearce-Levie K, Lewcock JW (2017). Loss of dual leucine zipper kinase signaling is protective in animal models of neurodegenerative disease. Sci Transl Med.

[CR25] Lucibello FC, Ehlert F, Müller R (1991). Multiple interdependent regulatory sites in the mouse c- fos promoter determine basal level transcription: cell type-specific effects. Nucleic Acids Res.

[CR26] Malik AN, Vierbuchen T, Hemberg M, Rubin AA, Ling E, Couch CH, Stroud H, Spiegel I, Farh KK-H, Harmin DA, Greenberg ME (2014). Genome-wide identification and characterization of functional neuronal activity–dependent enhancers. Nat Neurosci.

[CR27] Maruyama T, Dougan SK, Truttmann MC, Bilate AM, Ingram JR, Ploegh HL (2015). Increasing the efficiency of precise genome editing with CRISPR-Cas9 by inhibition of nonhomologous end joining. Nat Biotechnol.

[CR28] Nihalani D, Wong H, Verma R, Holzman LB (2007). Src family kinases directly regulate JIP1 module dynamics and activation. Mol Cell Biol.

[CR29] O'Donnell A, Yang S-H, Sharrocks AD (2008). MAP kinase-mediated c-fos regulation relies on a histone acetylation relay switch. Mol Cell.

[CR30] Oetjen E, Blume R, Cierny I, Schlag C, Kutschenko A, Krätzner R, Stein R, Knepel W (2007). Inhibition of MafA transcriptional activity and human insulin gene transcription by interleukin-1β and mitogen-activated protein kinase kinase kinase in pancreatic islet beta cells. Diabetologia.

[CR31] Oetjen E, Lechleiter A, Blume R, Nihalani D, Holzman L, Knepel W (2006). Inhibition of membrane depolarisation-induced transcriptional activity of cyclic AMP response element binding protein (CREB) by the dual-leucine-zipper-bearing kinase in a pancreatic islet beta cell line. Diabetologia.

[CR32] Oetjen E, Lemcke T (2016). Dual leucine zipper kinase (MAP3K12) modulators: a patent review (2010–2015). Expert Opin Ther Pat.

[CR33] Patel S, Harris SF, Gibbons P, Deshmukh G, Gustafson A, Kellar T, Lin H, Liu X, Liu Y, Liu Y, Ma C, Scearce-Levie K, Ghosh AS, Shin YG, Solanoy H, Wang J, Wang B, Yin J, Siu M, Lewcock JW (2015). Scaffold-hopping and structure-based discovery of potent, selective, and brain penetrant N-(1H-Pyrazol-3-yl)pyridin-2-amine inhibitors of dual leucine zipper kinase (DLK, MAP3K12). J Med Chem.

[CR34] Phu DT, Wallbach M, Depatie C, Fu A, Screaton RA, Oetjen E (2011). Regulation of the CREB coactivator TORC by the dual leucine zipper kinase at different levels. Cell Signal.

[CR35] Plaumann S, Blume R, Börchers S, Steinfelder HJ, Knepel W, Oetjen E (2008). Activation of the dual-leucine-zipper-bearing kinase and induction of β-cell apoptosis by the immunosuppressive drug cyclosporin A. Mol Pharmacol.

[CR36] Pozniak CD, Sengupta Ghosh A, Gogineni A, Hanson JE, Lee SH, Larson JL, Solanoy H, Bustos D, Li H, Ngu H, Jubb AM, Ayalon G, Wu J, Scearce-Levie K, Zhou Q, Weimer RM, Kirkpatrick DS, Lewcock JW (2013). Dual leucine zipper kinase is required for excitotoxicity-induced neuronal degeneration. J Exp Med.

[CR37] Qiu Z, Ghosh A (2008). A calcium-dependent switch in a CREST-BRG1 complex regulates activity-dependent gene expression. Neuron.

[CR38] Ran FA, Hsu PD, Wright J, Agarwala V, Scott DA, Zhang F (2013). Genome engineering using the CRISPR-Cas9 system. Nat Protoc.

[CR39] Ray JD, Kener KB, Bitner BF, Wright BJ, Ballard MS, Barrett EJ, Hill JT, Moss LG, Tessem JS (2016). Nkx6.1-mediated insulin secretion and β-cell proliferation is dependent on upregulation of c-Fos. FEBS Lett.

[CR40] Roche E, Buteau J, Aniento I, Reig JA, Soria B, Prentki M (1999). Palmitate and oleate induce the immediate-early response genes c-fos and nur-77 in the pancreatic beta-cell line INS-1. Diabetes.

[CR41] Runkel L, Shaw PE, Herrera RE, Hipskind RA, Nordheim A (1991). Multiple basal promoter elements determine the level of human c-fos transcription. Mol Cell Biol.

[CR42] Shaulian E, Karin M (2002). AP-1 as a regulator of cell life and death. Nat Cell Biol.

[CR43] Shin JE, Ha H, Kim YK, Cho Y, DiAntonio A (2019). DLK regulates a distinctive transcriptional regeneration program after peripheral nerve injury. Neurobiol Dis.

[CR44] Stahnke M-J, Dickel C, Schröder S, Kaiser D, Blume R, Stein R, Pouponnot C, Oetjen E (2014). Inhibition of human insulin gene transcription and MafA transcriptional activity by the dual leucine zipper kinase. Cell Signal.

[CR45] Susini S, Roche E, Prentki M, Schlegel W (1998). Glucose and glucoincretin peptides synergize to induce c-fos, c-jun, junB, zif-268, and nur-77 gene expression in pancreatic β(INS-1) cells. FASEB J.

[CR46] Susini S, van Haasteren G, Li S, Prentki M, SCHLEGEL W,  (2000). Essentiality of intron control in the induction of c-fos by glucose and glucoincretin peptides in INS-1 β-cells. FASEB J.

[CR47] Tedeschi A, Bradke F (2013). The DLK signalling pathway–a double-edged sword in neural development and regeneration. EMBO Rep.

[CR48] Tenenbaum M, Plaisance V, Boutry R, Pawlowski V, Jacovetti C, Sanchez-Parra C, Ezanno H, Bourry J, Beeler N, Pasquetti G, Gmyr V, Dalle S, Kerr-Conte J, Pattou F, Hirai S-i, Regazzi R, Bonnefond A, Froguel P, Abderrahmani A (2020) The map3k12 (Dlk)/JNK3 signaling pathway is required for pancreatic beta-cell proliferation during postnatal development. Cell Mol Life Sci 78:287–298. 10.1007/s00018-020-03499-7 10.1007/s00018-020-03499-7 PMC11072213 32189007

[CR49] Wallbach M, Duque Escobar J, Babaeikelishomi R, Stahnke M-J, Blume R, Schröder S, Kruegel J, Maedler K, Kluth O, Kehlenbach RH, Miosge N, Oetjen E (2016). Distinct functions of the dual leucine zipper kinase depending on its subcellular localization. Cell Signal.

