# Supplementary material for: Increase of c-FOS promoter transcriptional activity by the dual leucine zipper kinase
Source: Naunyn Schmiedebergs Arch Pharmacol. 2023 Jan 26;396(6):1223–33. doi: 10.1007/s00210-023-02401-z (PMC10185614; doi:10.1007/s00210-023-02401-z)
Supplement: Supplementary file 1 — Supplementary file1 (DOCX 13 KB) [file 210_2023_2401_MOESM1_ESM.docx]

Table 1: Sequences of primers, sgRNA and repair template. The restriction enzyme sites are bold and underlined.

| **Primer** | **Sequence (5' to 3')** |
| --- | --- |
| -287 to +48_for | AGATCC**AAGCTT**TTTCCACGGCCTTTCC |
| -303 to +48_for | AGATCC**AAGCTT**CATCTGCGTCAGCAGG |
| -315 to +48_for | AGATCC**AAGCTT**TCCATATTAGGACATC |
| -339 to +48_for | AGATCC**AAGCTT**ATCCCTCCCCCCTTA |
| -348 to +48_for | AGATCC**AAGCTT**TTCCCGTCAATCCCT |
| c-fos 5' rev | TCAGAT**CTCGAG**ATCCAGATCTGGATCC |
|  |  |
| -711 to -53_rev | GATTCA**CTCGAG**GTGTAAACGTCACGG |
| -711 to -284_rev | GATTCA**CTCGAG**GAAACCTGCTGACGCA |
| -711 to -298_rev | GATTCA**CTCGAG**CAGATGTCCTAATATGG |
| -711 to -312_rev | GATTCA**CTCGAG**TGGGACATCCTGTGTAAG |
| -711 to -333_rev | GATTCA**CTCGAG**GGAGGGATTGACGGGAA |
| c-fos 3' for | CTCAGATCC**AAGCTT**GCATGCCTGCA |
|  |  |
| Specific sgRNA | GGAGGAAGTGGCTGTGAAGA*AGG* |
| Repair template | TCAGGGTGCTGTTTTCCTGGGTCGCTTCCATGGGGAGGAAGTGGCTGTG**GCC**AA***A***GTTCGAGACCTCAAGGAGACCGACATCAAGCATCTGCGAAAGCTG |
